# Hyaluronan and synovial joint: function, distribution and healing

**DOI:** 10.2478/intox-2013-0019

**Published:** 2013-09

**Authors:** Tamer Mahmoud Tamer

**Affiliations:** 1 Polymer Materials Research Department, Advanced Technologies and New Materials Research Institute (ATNMRI), City of Scientific Research and Technological Applications (SRTA-City), New Borg El-Arab City, Alexandria, Egypt; 2 Laboratory of Bioorganic Chemistry of Drugs, Institute of Experimental Pharmacology & Toxicology, Slovak Academy of Sciences, Bratislava, Slovak Republic

**Keywords:** synovial joint fluid, hyaluronan, antioxidant, thiol compound

## Abstract

Synovial fluid is a viscous solution found in the cavities of synovial joints. The principal role of synovial fluid is to reduce friction between the articular cartilages of synovial joints during movement. The presence of high molar mass hyaluronan (HA) in this fluid gives it the required viscosity for its function as lubricant solution. Inflammation oxidation stress enhances normal degradation of hyaluronan causing several diseases related to joints.

This review describes hyaluronan properties and distribution, applications and its function in synovial joints, with short review for using thiol compounds as antioxidants preventing HA degradations under inflammation conditions.

## Introduction

**Figure 1:**
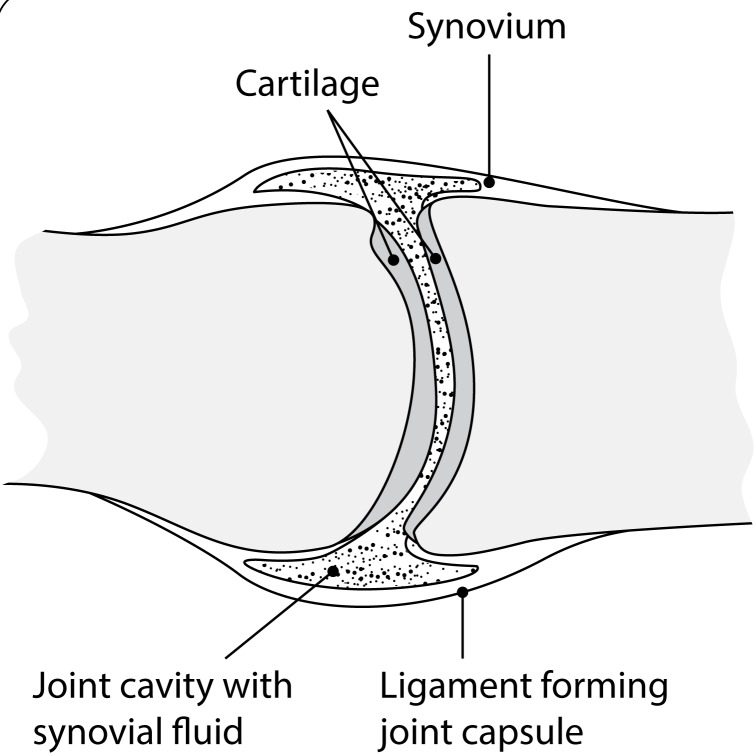
Normal, healthy synovial joint (adapted from Kogan, 2010).

The human skeleton consists of both fused and individual bones supported and supplemented by ligaments, tendons, and skeletal muscles. Articular ligaments and tendons are the main parts holding together the joint(s). In respect of movement, there are freely moveable, partially moveable, and immovable joints. Synovial joints (Figure 1), the freely moveable ones, allow for a large range of motion and encompass wrists, knees, ankles, shoulders, and hips (Kogan, 2010).

## Structure of synovial joints

### Cartilage

In a healthy synovial joint, heads of the bones are encased in a smooth (hyaline) cartilage layer. These tough slippery layers – *e.g.* those covering the bone ends in the knee joint – belong to mechanically highly stressed tissues in the human body. At walking, running, or sprinting the strokes frequency attain approximately 0.5, 2.5 or up to 10 Hz.

**Figure 2:**
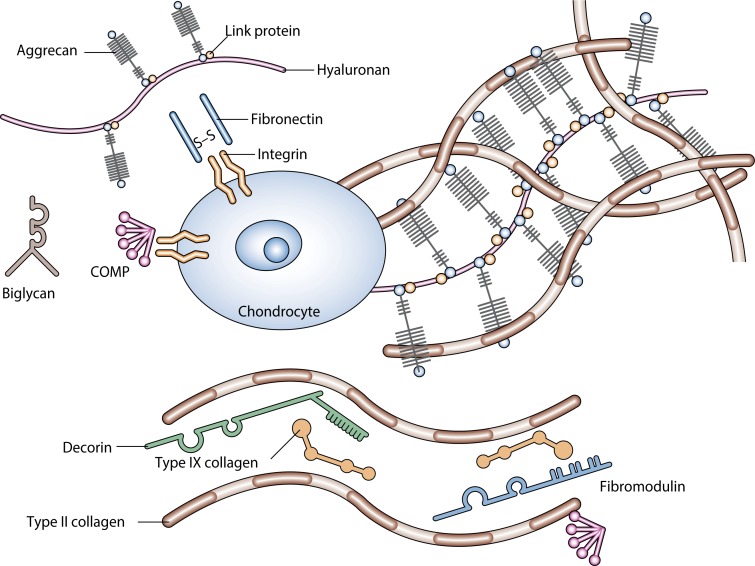
Articular cartilage main components and structure (adapted from Chen *et al.*, 2006).

Cartilage functions also as a shock absorber. This property is derived from its high water entrapping capacity as well as from the structure and intermolecular interactions among polymeric components that constitute the cartilage tissue (Servaty *et al.,* 2000). Figure 2 sketches a section of the cartilage – a chondrocyte cell that permanently restructures/rebuilds its extracellular matrix. Three classes of proteins exist in articular cartilage: collagens (mostly type II collagen); proteoglycans (primarily aggrecan); and other noncollagenous proteins (including link protein, fibronectin, COMP – cartilage oligomeric matrix protein) and the smaller proteoglycans (biglycan, decorin, and fibromodulin). The interaction between highly negatively charged cartilage proteoglycans and type II collagen fibrils is responsible for the compressive and tensile strength of the tissue, which resists applied load *in vivo*.

### Synovium/synovial membrane

Each synovial joint is surrounded by a fibrous, highly vascular capsule/envelope called synovium, whose internal surface layer is lined with a synovial membrane. Inside this membrane, type B synoviocytes (fibroblast-like cell lines) are localized/embedded. Their primary function is to continuously extrude high-molar-mass hyaluronans (HAs) into synovial fluid.

### Synovial fluid

The synovial fluid (SF) of natural joints normally functions as a biological lubricant as well as a biochemical pool through which nutrients and regulatory cytokines traverse. SF contains molecules that provide low-friction and low-wear properties to articulating cartilage surfaces.

Molecules postulated to play a key role in lubrication alone or in combination, are proteoglycan 4 (PRG4) (Swann *et al.,* 1985) present in SF at a concentration of 0.05–0.35 mg/ml (Schmid *et al.,* 2001), hyaluronan (HA) (Ogston & Stanier, 1953) at 1–4 mg/ml (Mazzucco *et al.,* 2004), and surface-active phospholipids (SAPL) (Schwarz & Hills, 1998) at 0.1 mg/ml (Mazzucco *et al.,* 2004). Synoviocytes secrete PRG4 (Jay *et al.,* 2000; Schumacher *et al.,* 1999) and are the major source of SAPL (Dobbie *et al.,* 1995; Hills & Crawford, 2003; Schwarz & Hills, 1996), as well as HA **(**Haubeck *et al.,* 1995; Momberger *et al.,* 2005) in SF. Other cells also secrete PRG4, including chondrocytes in the superficial layer of articular cartilage (Schmid *et al.,* 2001b; Schumacher *et al.,* 1994) and, to a much lesser extent, cells in the meniscus (Schumacher *et al.,* 2005).

As a biochemical depot, SF is an ultra filtrate of blood plasma that is concentrated by virtue of its filtration through the synovial membrane. The synovium is a thin lining (∼50 µm in humans) comprised of tissue macrophage A cells, fibroblast-like B cells (Athanasou & Quinn, 1991; Revell, 1989; Wilkinson *et al.,* 1992), and fenestrated capillaries (Knight & Levick, 1984). It is backed by a thicker layer (∼100 µm) of loose connective tissue called the subsynovium (SUB) that includes an extensive system of lymphatics for clearance of transported molecules. The cells in the synovium form a discontinuous layer separated by intercellular gaps of several microns in width (Knight & Levick, 1984; McDonald & Levick, 1988). The extracellular matrix in these gaps contains collagen types I, III, and V (Ashhurst *et al.,* 1991; Rittig *et al.,* 1992), hyaluronan (Worrall *et al.,* 1991), chondroitin sulphate (Price *et al.,* 1996; Worrall *et al.,* 1994), biglycan and decorin proteoglycans (Coleman *et al.,* 1998), and fibronectin (Poli *et al.,* 2004). The synovial matrix provides the permeable pathway through which exchange of molecules occurs (Levick, 1994), but also offers sufficient outflow resistance (Coleman *et al.,* 1998; Scott *et al.,* 1998) to retain large solutes of SF within the joint cavity. Together, the appropriate reflection of secreted lubricants by the synovial membrane and the appropriate lubricant secretion by cells are necessary for development of a mechanically functional SF (Blewis *et al.,* 2007).

**Figure 3:**
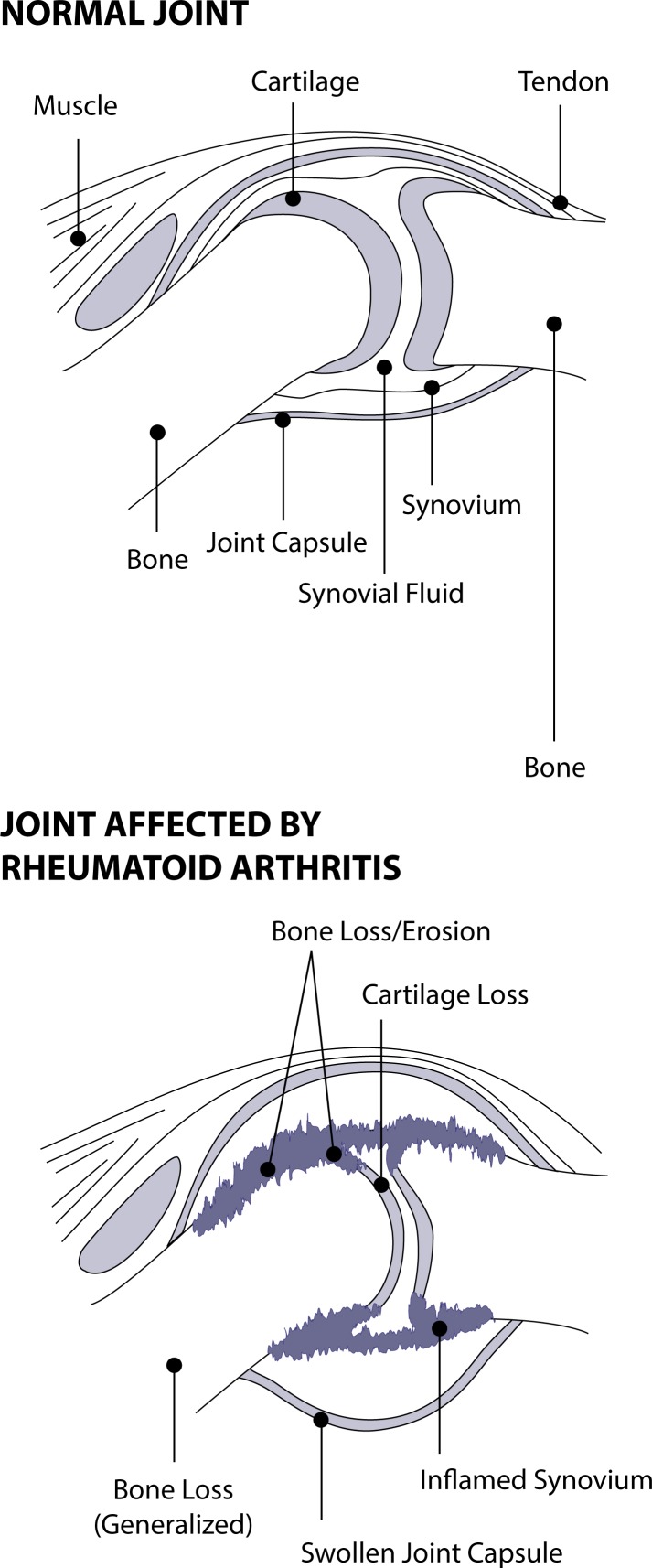
Normal, (healthy) and rheumatoid arthritis synovial joint.

In the joint, HA plays an important role in the protection of articular cartilage and the transport of nutrients to cartilage. In patients with rheumatoid arthritis (RA), (Figure 3) it has been reported that HA acts as an anti inflammatory substance by inhibiting the adherence of immune complexes to neutrophils through the Fc receptor (Brandt, 1970), or by protecting the synovial tissues from the attachment of inflammatory mediators (Miyazaki *et al.,* 1983, Mendichi & Soltes, 2002).

Reactive oxygen species (ROS) (O_2_^•–^, H_2_O_2_, ^•^OH) are generated in abundance by synovial neutrophils from RA patients, as compared with synovial neutrophils of osteoarthritis (OA) patients and peripheral neutrophils of both RA and OA patients (Niwa *et al.,* 1983).

McCord (1973) demonstrated that HA was susceptible to degradation by ROS *In vitro*, and that this could be protected by superoxide dismutase (SOD) and/or catalase, which suggests the possibility that there is pathologic oxidative damage to synovial fluid components in RA patients. Dahl *et al.* (1985) reported that there are reduced HA concentrations in synovial fluids from RA patients. It has also been reported that ROS scavengers inhibit the degradation of HA by ROS (Soltes, 2010; Blake *et al.,* 1981; Betts & Cleland, 1982; Soltes *et al.,* 2004).

These findings appear to support the hypothesis that ROS are responsible for the accelerated degradation of HA in the rheumatoid joint. In the study of Juranek and Soltes (2012) the oxygen radical scavenging activities of synovial fluids from both RA and OA patients were assessed, and the antioxidant activities of these synovial fluids were analyzed by separately examining HA, d-glucuronic acid, and *N*-acetyl-d-glucosamine.

## Hyaluronan

In 1934, Karl Meyer and his colleague John Palmer isolated a previously unknown chemical substance from the vitreous body of cows’ eyes. They found that the substance contained two sugar molecules, one of which was uronic acid. For convenience, therefore, they proposed the name “hyaluronic acid”. The popular name is derived from “hyalos”, which is the Greek word for glass + uronic acid (Meyer & Palmer, 1934)**.** At the time, they did not know that the substance which they had discovered would prove to be one of the most interesting and useful natural macromolecules. HA was first used commercially in 1942 when Endre Balazs applied for a patent to use it as a substitute for egg white in bakery products (Necas *et al.,* 2008).

The term “hyaluronan” was introduced in 1986 to conform to the international nomenclature of polysaccharides and is attributed to Endre Balazs (Balazs *et al.,* 1986) who coined it to encompass the different forms the molecule can take, e.g, the acid form, hyaluronic acid, and the salts, such as sodium hyaluronate, which forms at physiological pH (Laurent, 1989). HA was subsequently isolated from many other sources and the physicochemical structure properties and biological role of this polysaccharide were studied in numerous laboratories (Kreil, 1995). This work has been summarized in a Ciba Foundation Symposium (Laurent, 1989) and a recent review (Laurent & Fraser, 1992; Chabrecek *et al.,* 1990; Orvisky *et al.,* 1992).

**Figure 4:**
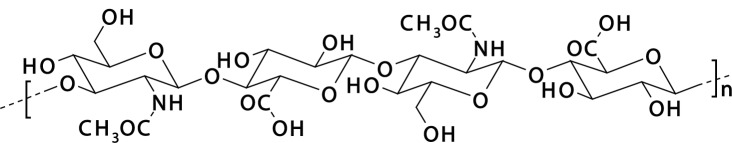
Structural formula of hyaluronan – the acid form.

Hyaluronan (Figure 4) is a unique biopolymer composed of repeating disaccharide units formed by *N*-acetyl-d-glucosamine and d-glucuronic acid. Both sugars are spatially related to glucose which in the β-configuration allows all of its bulky groups (the hydroxyls, the carboxylate moiety, and the anomeric carbon on the adjacent sugar) to be in sterically favorable equatorial positions while all of the small hydrogen atoms occupy the less sterically favorable axial positions. Thus, the structure of the disaccharide is energetically very stable. HA is also unique in its size, reaching up to several million Daltons and is synthesized at the plasma membrane rather than in the Golgi, where sulfated glycosaminoglycans are added to protein cores (Itano & Kimata, 2002; Weigel *et al.,* 1997; Kogan *et al.,* 2007a).

In a physiological solution, the backbone of a HA molecule is stiffened by a combination of the chemical structure of the disaccharide, internal hydrogen bonds, and interactions with the solvent. The axial hydrogen atoms form a non-polar, relatively hydrophobic face while the equatorial side chains form a more polar, hydrophilic face, thereby creating a twisting ribbon structure. Solutions of hyaluronan manifest very unusual rheological properties and are exceedingly lubricious and very hydrophilic. In solution, the hyaluronan polymer chain takes on the form of an expanded, random coil. These chains entangle with each other at very low concentrations, which may contribute to the unusual rheological properties. At higher concentrations, solutions have an extremely high but shear-dependent viscosity. A 1% solution is like jelly, but when it is put under pressure it moves easily and can be administered through a small-bore needle. It has therefore been called a “pseudo-plastic” material. The extraordinary rheological properties of hyaluronan solutions make them ideal as lubricants. There is evidence that hyaluronan separates most tissue surfaces that slide along each other. The extremely lubricious properties of hyaluronan have been shown to reduce postoperative adhesion formation following abdominal and orthopedic surgery. As mentioned, the polymer in solution assumes a stiffened helical configuration, which can be attributed to hydrogen bonding between the hydroxyl groups along the chain. As a result, a coil structure is formed that traps approximately 1000 times its weight in water (Chabrecek *et al.,* 1990; Cowman & Matsuoka, 2005; Schiller *et al.,* 2011)

## Properties of hyaluronan

### Hyaluronan networks

The physico-chemical properties of hyaluronan were studied in detail from 1950 onwards (Comper & Laurent, 1978).

The molecules behave in solution as highly hydrated randomly kinked coils, which start to entangle at concentrations of less than 1 mg/mL. The entanglement point can be seen both by sedimentation analysis (Laurent *et al.,* 1960) and viscosity (Morris *et al.,* 1980). More recently Scott and his group have given evidence that the chains when entangling also interact with each other and form stretches of double helices so that the network becomes mechanically more firm (Scott *et al.,* 1991).

### Rheological properties

Solutions of hyaluronan are viscoelastic and the viscosity is markedly shearing dependent (Morris *et al.,* 1980; Gibbs *et al.,* 1968). Above the entanglement point the viscosity increases rapidly and exponentially with concentration (∼c^3.3^) (Morris *et al.,* 1980) and a solution of 10 g/l may have a viscosity at low shear of ∼10^6^ times the viscosity of the solvent. At high shear the viscosity may drop as much as ∼10^3^ times (Gibbs *et al.,* 1968). The elasticity of the system increases with increasing molecular weight and concentration of hyaluronan as expected for a molecular network. The rheological properties of hyaluronan have been connected with lubrication of joints and tissues and hyaluronan is commonly found in the body between surfaces that move along each other, for example cartilage surfaces and muscle bundles (Bothner & Wik, 1987).

### Water homeostasis

A fixed polysaccharide network offers a high resistance to bulk flow of solvent (Comper & Laurent, 1978). This was demonstrated by Day (1950) who showed that hyaluronidase treatment removes a strong hindrance to water flow through a fascia. Thus HA and other polysaccharides prevent excessive fluid fluxes through tissue compartments. Furthermore, the osmotic pressure of a hyaluronan solution is non-ideal and increases exponentially with the concentration. In spite of the high molecular weight of the polymer the osmotic pressure of a 10 g/l hyaluronan solution is of the same order as an l0 g/l albumin solution. The exponential relationship makes hyaluronan and other polysaccharides excellent osmotic buffering substances – moderate changes in concentration lead to marked changes in osmotic pressure. Flow resistance together with osmotic buffering makes hyaluronan an ideal regulator of the water homeostasis in the body.

### Network interactions with other macromolecules

The hyaluronan network retards the diffusion of other molecules (Comper & Laurent, 1978; Simkovic *et al.,* 2000). It can be shown that it is the steric hindrance which restricts the movements and not the viscosity of the solution. The larger the molecule the more it will be hindered. *In vivo* hyaluronan will therefore act as a diffusion barrier and regulate the transport of other substances through the intercellular spaces. Furthermore, the network will exclude a certain volume of solvent for other molecules; the larger the molecule the less space will be available to it (Comper & Laurent, 1978). A solution of 10 g/l of hyaluronan will exclude about half of the solvent to serum albumin. Hyaluronan and other polysaccharides therefore take part in the partition of plasma proteins between the vascular and extravascular spaces. The excluded volume phenomenon will also affect the solubility of other macromolecules in the interstitium, change chemical equilibria and stabilize the structure of, for example, collagen fibers.

### Medical applications of hyaluronic acid

The viscoelastic matrix of HA can act as a strong biocompatible support material and is therefore commonly used as growth scaffold in surgery, wound healing and embryology. In addition, administration of purified high molecular weight HA into orthopaedic joints can restore the desirable rheological properties and alleviate some of the symptoms of osteoarthritis (Balazs & Denlinger, 1993; Balazs & Denlinger, 1989; Kogan *et al.,* 2007). The success of the medical applications of HA has led to the production of several successful commercial products, which have been extensively reviewed previously.

Table 1 summarizes both the medical applications and the commonly used commercial preparations containing HA used within this field. HA has also been extensively studied in ophthalmic, nasal and parenteral drug delivery. In addition, more novel applications including pulmonary, implantation and gene delivery have also been suggested. Generally, HA is thought to act as either a mucoadhesive and retain the drug at its site of action/absorption or to modify the *in vivo* release/absorption rate of the therapeutic agent. A summary of the drug delivery applications of HA is shown in Table 2.

**Table 1:** Summary of the medical applications of hyaluronic acid (Brown & Jones, 2005).

Disease state	Applications	Commercial products	Publications
Osteoarthritis	Lubrication and mechanical support for the joints	Hyalgan^®^ (Fidia, Italy) Artz^®^ (Seikagaku, Japan) ORTHOVISC^®^ (Anika, USA) Healon^®^, Opegan^®^ and Opelead^®^	Hochburg, 2000; Altman, 2000; Dougados, 2000; Guidolin *et al.,* 2001; Maheu *et al.,* 2002; Barrett & Siviero, 2002; Miltner *et al.,* 2002;Tascioglu and Oner, 2003; Uthman *et al.,* 2003; Kelly *et al.,* 2003; Hamburger *et al.,* 2003; Kirwan, 2001; Ghosh & Guidolin, 2002; Mabuchi *et al.,* 1999; Balazs, 2003; Fraser *et al.,* 1993; Zhu & Granick, 2003.
Surgery and wound healing	Implantation of artificial intraocular lens, viscoelastic gel	Bionect^®^, Connettivina^®^ and Jossalind^®^	Ghosh & Jassal, 2002; Risbert, 1997; Inoue & Katakami, 1993; Miyazaki *et al.,* 1996; Stiebel-Kalish *et al.,* 1998; Tani *et al.,* 2002; Vazquez *et al.,* 2003; Soldati *et al.,* 1999; Ortonne, 1996; Cantor *et al.,* 1998; Turino & Cantor, 2003.
Embryo implantation	Culture media for the use of *In vitro* fertilization	EmbryoGlue^®^ (Vitrolife, USA)	Simon *et al.,* 2003; Gardner *et al.,* 1999; Vanos *et al.,* 1991; Kemmann, 1998; Suchanek *et al.,* 1994; Joly *et al.,* 1992; Gardner, 2003; Lane *et al.,* 2003; Figueiredo *et al.,* 2002, Miyano *et al.,* 1994; Kano *et al.,* 1998; Abeydeera, 2002; Jaakma *et al.,* 1997; Furnus *et al.,* 1998;Jang *et al.,* 2003.

**Table 2:** Summary of the drug delivery applications of hyaluronic acid.

Route	Justification	Therapeutic agents	Publications
Ophthalmic	Increased ocular residence of drug, which can lead to increased bioavailability	Pilocarpine, tropicamide, timolol, gentimycin, tobramycin,arecaidine polyester, (S) aceclidine	Jarvinen *et al*., 1995; Sasaki *et al.,* 1996; Gurny *et al.,* 1987; Camber *et al.,* 1987; Camber & Edman, 1989; Saettone *et al.,* 1994; Saettone *et al.,* 1991; Bucolo *et al.,* 1998; Bucolo & Mangiafico, 1999; Herrero-Vanrell *et al.,* 2000; Moreira *et al.,* 1991; Bernatchez *et al.,* 1993; Gandolfi *et al.,* 1992 [Bibr CIT0050] 1997.
Nasal	Bioadhesion resulting in increased bioavailability	Xylometazoline, vasopressin, gentamycin	Morimoto *et al.,* 1991; Lim *et al.,* 2002.
Pulmonary	Absorption enhancer and dissolution rate modification	Insulin	Morimoto *et al.,* 2001; Surendrakumar *et al.,* 2003.
Parenteral	Drug carrier and facilitator of liposomal entrapment	Taxol, superoxide dismutase, human recombinant insulin-like growth factor, doxorubicin	Drobnik, 1991; Sakurai *et al.,* 1997; Luo and Prestwich, 1999; Luo *et al.,* 2000; Prisell *et al.,* 1992; Yerushalmi *et al.,* 1994; Yerushalmi & Margalit, 1998; Peer & Margalit, 2000; Eliaz & Szoka, 2001; Peer *et al.,* 2003.
Implant	Dissolution rate modification	Insulin	Surini *et al.,* 2003; Takayama *et al.,* 1990.
Gene	Dissolution rate modification and protection	Plasmid DNA/monoclonal antibodies	Yun *et al.,* 2004; Kim *et al.,* 2003.

### Cosmetic uses of hyaluronic acid

HA has been extensively utilized in cosmetic products because of its viscoelastic properties and excellent biocompatibility. Application of HA containing cosmetic products to the skin is reported to moisturize and restore elasticity, thereby achieving an antiwrinkle effect, albeit so far no rigorous scientific proof exists to substantiate this claim. HA-based cosmetic formulations or sunscreens may also be capable of protecting the skin against ultraviolet irradiation due to the free radical scavenging properties of HA (Manuskiatti & Maibach, 1996).

HA, either in a stabilized form or in combination with other polymers, is used as a component of commercial dermal fillers (*e.g.* Hylaform^®^, Restylane^®^ and Dermalive^®^) in cosmetic surgery. It is reported that injection of such products into the dermis, can reduce facial lines and wrinkles in the long term with fewer side-effects and better tolerability compared with the use of collagen (Duranti *et al.,* 1998; Bergeret-Galley *et al.,* 2001; Leyden *et al.,* 2003). The main side-effect may be an allergic reaction, possibly due to impurities present in HA (Schartz, 1997; Glogau, 2000).

## Biological function of hyaluronan

Naturally, hyaluronan has essential roles in body functions according to organ type in which it is distributed (Laurent *et al.,* 1996).

### Space filler

The specific functions of hyaluronan in joints are still essentially unknown. The simplest explanation for its presence would be that a flow of hyaluronan through the joint is needed to keep the joint cavity open and thereby allow extended movements of the joint. Hyaluronan is constantly secreted into the joint and removed by the synovium. The total amount of hyaluronan in the joint cavity is determined by these two processes. The half-life of the polysaccharide at steady-state is in the order of 0.5–1 day in rabbit and sheep (Brown *et al.,* 1991; Fraser *et al.,* 1993). The volume of the cavity is determined by the pressure conditions (hydrostatic and osmotic) in the cavity and its surroundings. Hyaluronan could, by its osmotic contributions and its formation of flow barriers in the limiting layers, be a regulator of the pressure and flow rate (McDonald & Leviek, 1995). It is interesting that in fetal development the formation of joint cavities is parallel with a local increase in hyaluronan (Edwards *et al.,* 1994).

### Lubrication

Hyaluronan has been regarded as an ideal lubricant in the joints due to its shear-dependent viscosity (Ogston & Stanier, 1953) but its role in lubrication has been refuted by others (Radin *et al.,* 1970). However, there are now reasons to believe that the function of hyaluronan is to form a film between the cartilage surfaces. The load on the joints may press out water and low-molecular solutes from the hyaluronan layer into the cartilage matrix. As a result, the concentration of hyaluronan increases and a gel structure of micrometric thickness is formed which protects the cartilage surfaces from frictional damage (Hlavacek, 1993). This mechanism to form a protective layer is much less effective in arthritis when the synovial hyaluronan has both a lower concentration and a lower molecular weight than normal. Another change in the arthritic joint is the protein composition of the synovial fluid. Fraser *et al.* (1972) showed more than 40 years ago that addition of various serum proteins to hyaluronan substantially increased the viscosity and this has received a renewed interest in view of recently discovered hyaladherins (see above). TSG-6 and inter-α-trypsin inhibitor and other acute phase reactants such as haptoglobin are concentrated to arthritic synovial fluid (Hutadilok *et al.,* 1988). It is not known to what extent these are affecting the rheology and lubricating properties.

### Scavenger functions

Hyaluronan has also been assigned scavenger functions in the joints. It has been known since the 1940s that hyaluronan is degraded by various oxidizing systems and ionizing irradiation and we know today that the common denominator is a chain cleavage induced by free radicals, essentially hydroxy radicals (Myint *et al.,* 1987). Through this reaction hyaluronan acts as a very efficient scavenger of free radicals. Whether this has any biological importance in protecting the joint against free radicals is unknown. The rapid turnover of hyaluronan in the joints has led to the suggestion that it also acts as a scavenger for cellular debris (Laurent *et al.,* 1995). Cellular material could be caught in the hyaluronan network and removed at the same rate as the polysaccharide (Stankovska *et al.,* 2007; Rapta, *et al.,* 2009).

### Regulation of cellular activities

As discussed above, more recently proposed functions of hyaluronan are based on its specific interactions with hyaladherins. One interesting aspect is the fact that hyaluronan influences angiogenesis but the effect is different depending on its concentration and molecular weight (Sattar *et al.,* 1992). High molecular weight and high concentrations of the polymer inhibit the formation of capillaries, while oligosaccharides can induce angiogenesis. There are also reports of hyaluronan receptors on vascular endothelial cells by which hyaluronan could act on the cells (Edwards *et al.,* 1995). The avascularity of the joint cavity could be a result of hyaluronan inhibition of angiogenesis.

Another interaction of some interest in the joint is the binding of hyaluronan to cell surface proteins. Lymphocytes and other cells may find their way to joints through this interaction. Injection of high doses of hyaluronan intra-articularly could attract cells expressing these proteins. Cells can also change their expression of hyaluronan-binding proteins in states of disease, whereby hyaluronan may influence immunological reactions and cellular traffic in the path of physiological processes in cells (Edwards *et al.,* 1995). The observation often reported that intra-articular injections of hyaluronan alleviate pain in joint disease (Adams, 1993) may indicate a direct or indirect interaction with pain receptors.

## Hyaluronan and synovial fluid

In normal/healthy joint, the synovial fluid, which consists of an ultrafiltrate of blood plasma and glycoproteins contains HA macromolecules of molar mass ranging between 6–10 mega Daltons (Praest *et al.,* 1997). SF serves also as a lubricating and shock absorbing boundary layer between moving parts of synovial joints. SF reduces friction and wear and tear of the synovial joint playing thus a vital role in the lubrication and protection of the joint tissues from damage during motion (Oates *et al.,* 2002).

As SF of healthy humans exhibits no activity of hyaluronidase, it has been inferred that oxygen-derived free radicals are involved in a self-perpetuating process of HA catabolism within the joint (Grootveld *et al.,* 1991; Stankovska *et al.,* 2006; Rychly *et al.,* 2006). This radical-mediated process is considered to account for ca. twelve-hour half-life of native HA macromolecules in SF.

**Figure 5:**
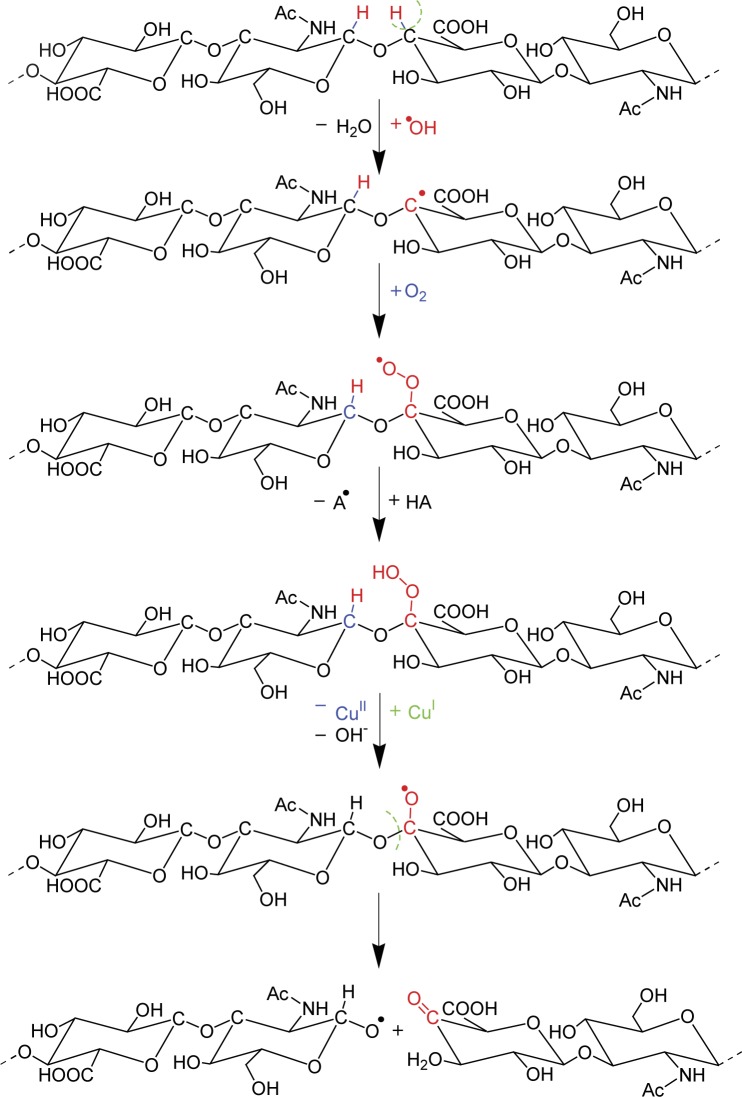
Schematic degradation of HA under free radical stress (Hrabarova *et al.,* 2012).

Acceleration of degradation of high-molecular-weight HA occurring under inflammation and/or oxidative stress is accompanied by impairment and loss of its viscoelastic properties (Parsons *et al.,* 2002; Soltes *et al.,* 2005; Stankovska *et al.,* 2005; Lath *et al.,* 2005; Hrabarova *et al.,* 2007; Valachova & Soltes, 2010; Valachova *et al.,* 2013a). Low-molecular weight HA was found to exert different biological activities compared to the native high-molecular-weight biopolymer. HA chains of 25–50 disaccharide units are inflammatory, immune-stimulatory, and highly angiogenic. HA fragments of this size appear to function as endogenous danger signals, reflecting tissues under stress (Noble, 2002; West *et al.,* 1985; Soltes *et al.,* 2007; Stern *et al.,* 2007; Soltes & Kogan, 2009). Figure 5 describes the fragmentation mechanism of HA under free radical stress.

**Figure 6:**
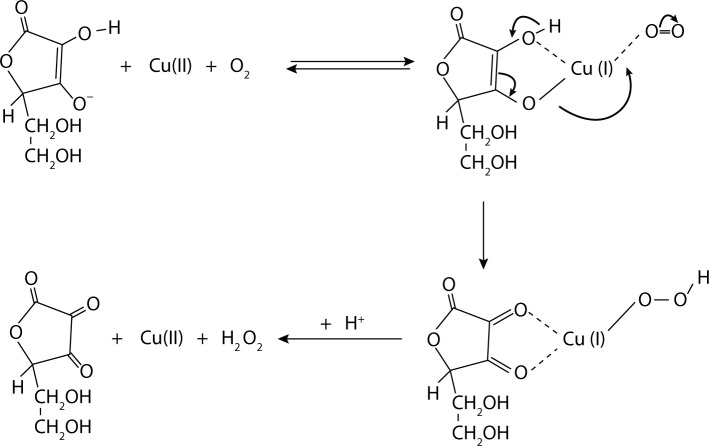
Scheme. Generation of H_2_O_2_ by Weissberger's system from ascorbate and Cu(II) ions under aerobic conditions (Valachova *et al.,* 2011)

- Initiation phase: the intact hyaluronan macromolecule entering the reaction with the HO^•^ radical formed via the Fenton-like reaction:
- Cu^+^ + H_2_O_2_ → Cu^2+^ + HO^•^ + OH^–^
- H_2_O_2_ has its origin due to the oxidative action of the Weissberger system (see Figure 6)
- Formation of an alkyl radical (C-centered hyaluronan macroradical) initiated by the HO^•^ radical attack.
- Propagation phase: formation of a peroxy-type C-macroradical of hyaluronan in a process of oxygenation after entrapping a molecule of O_2_.
- Formation of a hyaluronan-derived hydroperoxide via the reaction with another hyaluronan macromolecule.
- Formation of highly unstable alkoxy-type C-macroradical of hyaluronan on undergoing a redox reaction with a transition metal ion in a reduced state.
- Termination phase: quick formation of alkoxy-type C-fragments and the fragments with a terminal C=O group due to the glycosidic bond scission of hyaluronan. Alkoxy-type C fragments may continue the propagation phase of the free-radical hyaluronan degradation reaction. Both fragments are represented by reduced molar masses (Kogan, 2011; Rychly *et al.,* 2006; Hrabarova *et al.,* 2012; Surovcikova *et al.,* 2012; Valachova *et al.,* 2013b; Banasova *et al.,* 2012).

Several thiol compounds have attracted much attention from pharmacologists because of their reactivity toward endobiotics such as hydroxyl radical-derived species. Thiols play an important role as biological reductants (antioxidants) preserving the redox status of cells and protecting tissues against damage caused by the elevated reactive oxygen/nitrogen species (ROS/RNS) levels, by which oxidative stress might be indicated.

Soltes and his coworkers examined the effect of several thiol compounds on inhibition of the degradation kinetics of a high-molecular-weight HA *In vitro*. High molecular weight hyaluronan samples were exposed to free-radical chain degradation reactions induced by ascorbate in the presence of Cu(II) ions, the so called Weissberger's oxidative system. The concentrations of both reactants [ascorbate, Cu(II)] were comparable to those that may occur during an early stage of the acute phase of joint inflammation (see Figure 6) (Banasova *et al.,* 2011; Valachova *et al.,* 2011; Soltes *et al.,* 2006a; Soltes *et al.,* 2006b; Stankovska *et al.,* 2004; Soltes *et al.,* 2006c; Soltes *et al.,* 2007; Valachova *et al.,* 2008; 2009; 2010; 2011; 2013; Hrabarova *et al.,* 2009, 2011; Rapta *et al.,* 2009; 2010; Surovcikova-Machova *et al.,* 2012; Banasova *et al.,* 2011; Drafi *et al.,* 2010; Fisher & Naughton, 2005).

**Figure 7:**
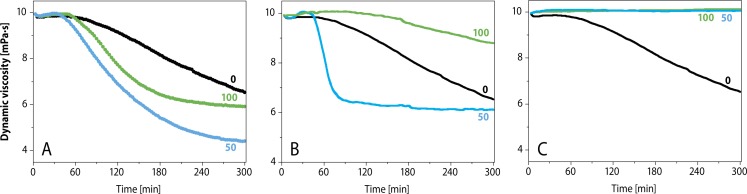
Effect of **A)** l-penicillamine, **B)** l-cysteine and **C)** bucillamine with different concentrations (50, 100 µM) on HA degradation induced by the oxidative system containing 1.0 µM CuCl_2_ + 100 µM ascorbic acid (Valachova *et al.,* 2011).

Figure 7 illustrates the dynamic viscosity of hyaluronan solution in the presence and absence of bucillamine, d-penicillamine and l-cysteine as inhibitors for free radical degradation of HA. The study showed that bucillamine to be both a preventive and chain-breaking antioxidant. On the other hand, d-penicillamine and l-cysteine dose dependently act as scavenger of ^•^OH radicals within the first 60 min. Then, however, the inhibition activity is lost and degradation of hyaluronan takes place (Valachova *et al.,* 2011; Valachova *et al.,* 2009; 2010; Hrabarova *et al.,* 2009).

l-Glutathione (GSH; l-γ-glutamyl-l-cysteinyl-glycine; a ubiquitous endogenous thiol, maintains the intracellular reduction-oxidation (redox) balance and regulates signaling pathways during oxidative stress/conditions. GSH is mainly cytosolic in the concentration range of ca. 1–10 mM; however, in the plasma as well as in SF, the range is only 1–3 µM (Haddad & Harb, 2005). This unique thiol plays a crucial role in antioxidant defense, nutrient metabolism, and in regulation of pathways essential for the whole body homeostasis. Depletion of GSH results in an increased vulnerability of the cells to oxidative stress (Hultberg & Hultberg, 2006).

It was found that l-glutathione exhibited the most significant protective and chain-breaking antioxidative effect against hyaluronan degradation. Thiol antioxidative activity, in general, can be influenced by many factors such as various molecule geometry, type of functional groups, radical attack accessibility, redox potential, thiol concentration and pK_a_, pH, ionic strength of solution, as well as different ability to interact with transition metals (Hrabarova *et al.,* 2012).

**Figure 8:**
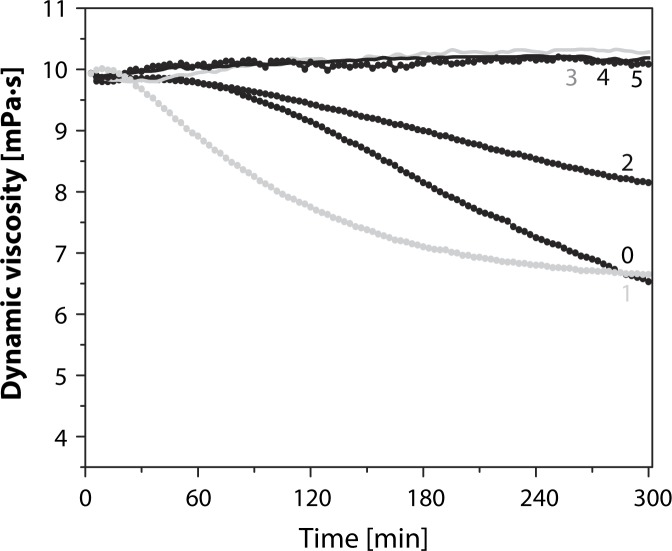
Comparison of the effect of l-glutathione on HA degradation induced by the system containing 1.0 µM CuCl_2_ plus 100 µM l-ascorbic acid. Concentration of l-glutathione in µM: 1–1.0; 2–10; 3, 4, 5–50, 100, and 200. Concentration of reference experiment: 0–nil thiol concentration (Hrabarova *et al.,* 2009; Valachova *et al.,* 2010a).

Figure 8 shows the dynamic viscosity versus time profiles of HA solution stressed to degradation with Weissberger's oxidative system. As evident, addition of different concentrations of GSH resulted in a marked protection of the HA macromolecules against degradation. The greater the GSH concentration used, the longer was the observed stationary interval in the sample viscosity values. At the lowest GSH concentration used, *i.e.* 1.0 µM (Figure 8), the time-dependent course of the HA degradation was more rapid than that of the reference experiment with the zero thiol concentration. Thus, one could classify GSH traces as functioning as a pro-oxidant.

**Figure 9:**
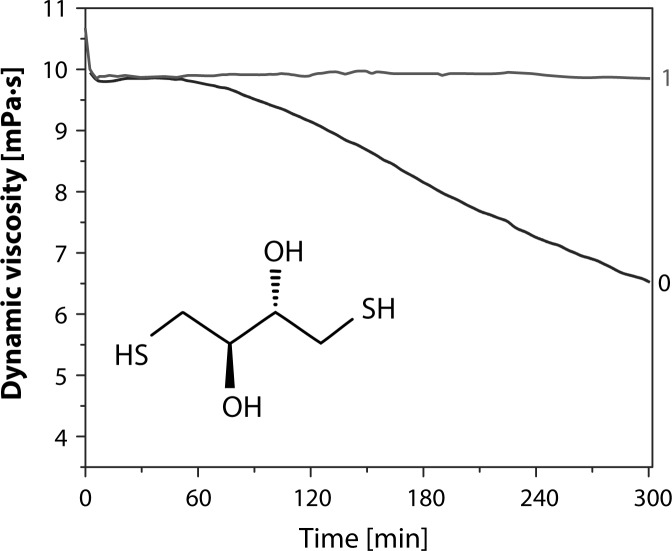
Effect of 1,4-dithioerythritol (1) on HA degradation induced by Weissberger's oxidative system (0) (Hrabarova *et al.,* 2010).

The effectiveness of antioxidant activity of 1,4-dithioerythritol expressed as the radical scavenging capacity was studied by a rotational viscometry method (Hrabarova *et al.,* 2010). 1,4-dithioerythritol, widely accepted and used as an effective antioxidant in the field of enzyme and protein oxidation, is a new potential antioxidant standard exhibiting very good solubility in a variety of solvents. Figure 9 describes the effect of 1,4-dithioerythritol on degradation of HA solution under free radical stress (Hrabarova *et al.,* 2010).

*N*-Acetyl-l-cysteine (NAC), another significant precursor of the GSH biosynthesis, has broadly been used as effective antioxidant in a form of nutritional supplement (Soloveva *et al.,* 2007; Thibodeau *et al.,* 2001). At low concentrations, it is a powerful protector of α_1_-antiproteinase against the enzyme inactivation by HOCl. NAC reacts with HO^•^ radicals and slowly with H_2_O_2_; however, no reaction of this endobiotic with superoxide anion radical was detected (Aruoma *et al.,* 1989).

**Figure 10:**
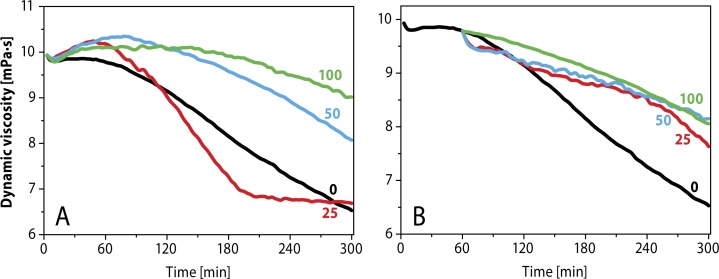
Evaluation of antioxidative effects of *N*-acetyl-l-cysteine against high-molar-mass hyaluronan degradation *in vitro* induced by Weissberger′s oxidative system. Reference sample (black): 1 M Cu(II) ions plus 100 µM ascorbic acid; nil thiol concentration. *N*-Acetyl-l-cysteine addition at the onset of the reaction (A) and after 1 h (B) (25, 50,100 µM). (Hrabarova *et al.,* 2012).

Investigation of the antioxidative effect of *N*-Acetyl-l-cysteine. Unlike l-glutathione, *N*-acetyl-l-cysteine was found to have preferential tendency to reduce Cu(II) ions to Cu(I), forming *N*-acetyl-l-cysteinyl radical that may subsequently react with molecular O_2_ to give O_2_^•–^ (Soloveva *et al.,* 2007; Thibodeau *et al.,* 2001). Contrary to l-cysteine, NAC (25 and 50 µM), when added at the beginning of the reaction, exhibited a clear antioxidative effect within ca. 60 and 80 min, respectively (Figure 10A). Subsequently, NAC exerted a modest pro-oxidative effect, more profound at 25-µM than at 100-µM concentration (Figure 10A). Application of NAC 1 h after the onset of the reaction (Figure 10B) revealed its partial inhibitory effect against formation of the peroxy-type radicals, independently from the concentration applied (Hrabarova *et al.,* 2012).

An endogenous amine, cysteamine (CAM) is a cystine-depleting compound with antioxidative and anti-inflammatory properties; it is used for treatment of cystinosis – a metabolic disorder caused by deficiency of the lysosomal cystine carrier. CAM is widely distributed in organisms and considered to be a key regulator of essential metabolic pathways (Kessler *et al.,* 2008).

**Figure 11:**
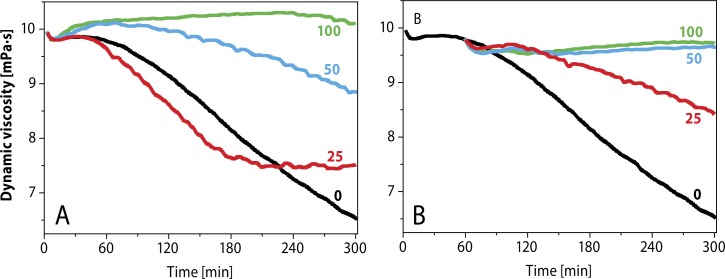
Evaluation of antioxidative effects of cysteamine against high-molar-mass hyaluronan degradation *in vitro* induced by Weissberger′s oxidative system. Reference sample (black): 1 mM CuII ions plus 100µM ascorbic acid; nil thiol concentration. Cysteamine addition at the onset of the reaction (a) and after 1 h (b) (25, 50,100 µM). (Hrabarova *et al.,* 2012).

Investigation of the antioxidative effect of cysteamine. Cysteamine (100 µM), when added before the onset of the reaction, exhibited an antioxidative effect very similar to that of GSH (Figure 8A and Figure 11A). Moreover, the same may be concluded when applied 1 h after the onset of the reaction (Figure 11B) at the two concentrations (50 and 100 µM), suggesting that CAM may be an excellent scavenger of peroxy radicals generated during the peroxidative degradation of HA (Hrabarova *et al.,* 2012).
